# Preparation-Route-Associated Differences in Color and Non-Volatile Metabolite Profiles of *Eurotium cristatum*-Fermented Fu Tea

**DOI:** 10.3390/foods15152719

**Published:** 2026-08-01

**Authors:** Yan Wang, Lisha Zhen, Yan Li, Shanshan Han

**Affiliations:** 1 Shaanxi Institute of Microbiology, Shaanxi Key Laboratory of Qinling Ecological Security, Shaanxi Academy of Sciences, Xi’an 710043, China; wangy@xab.ac.cn (Y.W.); zhenls@xab.ac.cn (L.Z.); liyanwsw@126.com (Y.L.); 2 Bio-Agriculture Institute of Shaanxi Province, Shaanxi Academy of Sciences, Xi’an 710043, China

**Keywords:** Fu tea, *Eurotium cristatum*, liquid-state fermentation, solid-state fermentation, non-volatile metabolites, tea pigments, free amino acids, flavonoids

## Abstract

Liquid- and solid-state fermentation are both used to produce *Eurotium cristatum*-fermented Fu tea, but their final soluble products have rarely been compared directly. We compared a liquid-state fermented tea infusion (LFt-D7) with an aqueous extract of solid-state fermented Fu tea (SFt-D7), using an unfermented sterilized tea extract (SHt-D0) as the reference group. LFt-D7 developed a darker reddish-brown appearance and had higher theaflavin and theabrownin contents, lower thearubigin content, and larger Commission Internationale de l’Éclairage L*a*b* (CIELAB) color differences than SHt-D0 and SFt-D7. Liquid chromatography–tandem mass spectrometry (LC–MS/MS) profiling separated the three endpoint groups; 221, 180, and 203 Level 2 annotations met the variable importance in projection (VIP > 1) and nominal *p* < 0.05 screening criteria in LFt-D7 versus SHt-D0, SFt-D7 versus SHt-D0, and LFt-D7 versus SFt-D7, respectively. Targeted analyses showed substantially lower free amino acid levels in SFt-D7. LFt-D7 retained a larger soluble free-amino-acid pool, had the highest total quantified flavonoid content with a stronger flavanol contribution, and showed higher relative peak-intensity signals for selected alkaloid- and phenolic acid-related metabolites. Mantel analysis showed covariation between metabolite modules and pigment indices, CIELAB parameters, total free amino acid (FAA) content, and total quantified flavonoid content. Together, these results show that the two complete preparation routes produce different soluble chemical profiles.

## 1. Introduction

Fu tea is a representative post-fermented dark tea in China, and its characteristic appearance and taste-associated chemical profile are closely related to microbial fermentation. During the traditional “flowering” process, *Eurotium cristatum* (*E. cristatum*) is one of the key fungi associated with the formation of “golden flowers” and with the biochemical transformation of tea-derived substrates [1,2]. Through microbial enzymatic activity and fermentation-associated physicochemical reactions, tea pigments, phenolic compounds, amino acid-related metabolites, alkaloids, sugars, and other non-volatile constituents can be substantially reshaped [2,3,4]. These coordinated changes form the chemical basis of the distinctive color and taste-associated attributes of Fu tea, although perceived sensory quality depends on the combined contribution of multiple compounds.

Traditional Fu tea is mainly produced through solid-state fermentation, in which microorganisms grow on moistened tea leaves under controlled temperature and humidity. This substrate-bound process has been studied extensively in relation to microbial succession, metabolite conversion, volatile formation, and quality-related chemical changes [1,4,5,6]. By contrast, liquid-state or submerged fermentation of tea infusions has attracted increasing attention as a route to fermented beverages and instant tea products [7,8]. The two routes differ in fermentation matrix, substrate accessibility, thermal treatment, and extraction history, and their soluble end products integrate all of these processing steps.

Color and taste-associated chemical traits are commonly used to characterize fermented tea. Tea pigments, including theaflavins, thearubigins, and theabrownins, contribute to infusion color and reflect the relative proportions of yellow, red and brown components [9,10,11]. Other non-volatile constituents, including free amino acids, flavonoids, phenolic acids, organic acids, sugars, and alkaloids, are related to umami, sweetness, bitterness, astringency, and phenolic-derived aftertaste in tea infusions [3,12,13]. These metabolite classes respond strongly to tea processing and *E. cristatum*-mediated fermentation [5,6,7,14]. Their combined evaluation can therefore provide a more complete account of endpoint chemical differentiation.

Previous metabolomic studies have examined compositional changes during solid-state [5,6] or liquid-state *E. cristatum* fermentation [7,8]. Recent reviews have outlined the wider application of metabolomics to tea quality assessment and processing research [15,16], while dark teas prepared from the same raw material through different microbial processes have also been compared [14]. Direct comparisons between a liquid-state fermented tea infusion and an aqueous extract of solid-state fermented tea remain limited, particularly when the raw material and fermentation duration are matched.

Here, we compared a liquid-state fermented tea infusion with an aqueous extract of solid-state fermented tea prepared from the same batch of dark tea over a 7-day fermentation period. Tea pigments and Commission Internationale de l’Éclairage L*a*b* (CIELAB) color attributes were measured together with free amino acids, targeted flavonoids, and untargeted non-volatile metabolites to characterize route-associated differences in the soluble products.

## 2. Materials and Methods

### 2.1. Raw Material and Fungal Inoculum

Dark tea was obtained from Xianyang Jingwei Fu Tea Co., Ltd. (Xianyang, China), and the same batch was used for both preparation routes. The fungal isolate EC-JW01 was obtained from the production culture used at the same company and was maintained at −80 °C in our laboratory. The isolate was characterized by colony morphology and bidirectional Sanger sequencing of the internal transcribed spacer (ITS) region using the primers ITS1 (5′-TCCGTAGGTGAACCTGCGG-3′) and ITS4 (5′-TCCTCCGCTTATTGATATGC-3′). Primer synthesis and bidirectional Sanger sequencing were performed by Beijing Tsingke Biotech Co., Ltd. (Beijing, China). The forward and reverse reads were assembled into a 526-bp consensus sequence (GenBank accession PZ742006). BLASTn analysis using the NCBI BLASTn web server (BLAST+ version 2.17.0; National Center for Biotechnology Information, Bethesda, MD, USA) showed 100% identity across a 520-bp aligned region with the type-strain sequence of *Aspergillus cristatus* (=*E*. *cristatum*) NRRL 4222 (GenBank accession NR_135341.1), supporting its identification as *E. cristatum*. Representative colony morphology is shown in Figure S1.

For inoculum preparation, the isolate was cultured on potato dextrose agar (PDA, Beijing Aoboxing Biotechnology Co., Ltd., Beijing, China; Cat. No. 02-023) at 28 °C for 5 days. Spores were harvested by washing the culture surface with sterile water, and the suspension was filtered through sterile gauze to remove mycelial fragments. Spore concentration was determined using a hemocytometer (Paul Marienfeld GmbH & Co. KG, Lauda-Königshofen, Germany) under a CX43 light microscope (Olympus Corporation, Tokyo, Japan) and adjusted to 1 × 10^6^ spores/mL with sterile water. Suspensions prepared in this manner were used for both liquid- and solid-state fermentation.

### 2.2. Experimental Design and Preparation Routes

Three endpoint groups were prepared from the same batch of dark raw tea using a 7-day fermentation period: SHt-D0, an unfermented sterilized aqueous tea extract; LFt-D7, the tea infusion after 7 days of liquid-state fermentation; and SFt-D7, the aqueous extract of solid-state fermented Fu tea after 7 days of flowering.

For the liquid-state route, dark raw tea was mixed with deionized water at a solid-to-liquid ratio of 1:25 (*w*/*v*) and sterilized at 115 °C for 20 min to obtain a controlled aqueous tea matrix. After cooling to room temperature, part of the sterilized aqueous extract was collected as SHt-D0. The remaining sterilized aqueous extract was inoculated with *E. cristatum* spore suspension and fermented for 7 days to obtain LFt-D7. Liquid-state fermentation was conducted in sterile 250 mL Erlenmeyer flasks containing 50 mL of sterilized tea extract. Each flask was inoculated with 5 mL of spore suspension (1 × 10^6^ spores/mL), providing 5 × 10^6^ spores per flask, and incubated at 37 °C for 7 days with shaking at 150 rpm.

For the solid-state route, 100 mL of the same spore suspension (1 × 10^6^ spores/mL) was used per kilogram of final substrate. The suspension was included in the sterile water used to adjust the initial moisture content to approximately 40% (*w*/*w*, wet basis), giving a nominal inoculum level of 1 × 10^5^ spores/g. After thorough mixing, the inoculated tea was divided into six 500-g portions and mechanically pressed in a rectangular mold into bricks measuring approximately 15 × 10 × 3 cm. Each brick was wrapped separately in sterilized kraft paper and fermented at 28 °C and 70% relative humidity for 7 days under natural ventilation. The bricks were turned once daily, and no additional water was supplied during fermentation. Each brick was subsequently ground and extracted separately with deionized water at 1:25 (*w*/*v*) and 100 °C for 20 min.

Each group comprised six independently prepared experimental units. SHt-D0 consisted of six independently prepared sterilized tea extracts, LFt-D7 of six independently inoculated fermentation flasks, and SFt-D7 of six independently fermented and extracted tea bricks. All samples were stored at −80 °C until physicochemical and metabolomic analyses.

### 2.3. Determination of Tea Pigments and CIELAB Color Attributes

Color-associated physicochemical traits were evaluated by combining tea pigment quantification with CIELAB color analysis. Spectrophotometric measurements were performed using a 721 visible spectrophotometer (Shanghai Xinmao, Shanghai, China) with 1 cm cuvettes. The instrument was equipped with a tungsten lamp and operated over a wavelength range of 350–1020 nm with a spectral bandwidth of 6 nm.

Tea pigments were quantified using solvent-partitioning spectrophotometric assays. Thearubigins and theabrownins were determined according to NY/T 3675-2020 [17]. For each sample, three colorimetric fractions were prepared. Fraction A was prepared by mixing 2 mL of tea extract with 2 mL of saturated oxalic acid solution and 6 mL of distilled water, followed by dilution to 25 mL with 95% ethanol. Fraction B was prepared by extracting 10 mL of tea extract with 10 mL of *n*-butanol for 5 min. After phase separation, 2 mL of the lower aqueous phase was collected, mixed with 2 mL of saturated oxalic acid solution and 6 mL of distilled water, and diluted to 25 mL with 95% ethanol. Fraction C was prepared by extracting 15 mL of tea extract with 15 mL of ethyl acetate for 5 min. After phase separation, 6 mL of the upper ethyl acetate phase was mixed with 6 mL of 25 g/L sodium bicarbonate solution, shaken for 30 s, and allowed to separate; 4 mL of the resulting upper ethyl acetate phase was then diluted to 25 mL with 95% ethanol. Absorbance values of fractions A, B and C were recorded at 380 nm using 95% ethanol as the reference, and thearubigin and theabrownin contents were calculated according to the standard method.

Theaflavins were quantified using an ethyl acetate/sodium bicarbonate partitioning assay. Briefly, 15 mL of each endpoint sample was extracted with 15 mL of ethyl acetate for 5 min. After phase separation, 8 mL of the upper ethyl acetate phase was mixed with 8 mL of 25 g/L sodium bicarbonate solution, shaken for 30 s, and allowed to separate. Subsequently, 4 mL of the upper ethyl acetate phase was diluted to 25 mL with 95% ethanol. Absorbance was recorded at 380 nm using 95% ethanol as the reference, and theaflavin content was calculated after correction for dry matter content. The contents of all tea pigment groups were calculated with correction for dry matter content and expressed as percentages (%).

CIELAB color attributes were calculated from visible-range transmittance data acquired with the same spectrophotometer using distilled water as the reference. The L*, a* and b* values were calculated under CIE standard illuminant D_65_ and 10° observer conditions according to the CIE 1976 L*a*b* color space [18]. Chroma was calculated as C*_ab_ = [(a*)^2^ + (b*)^2^]^1/2^. The total color difference was calculated as ΔE*_ab_ = [(ΔL*)^2^ + (Δa*)^2^ + (Δb*)^2^]^1/2^, where ΔL*, Δa* and Δb* represent pairwise differences in L*, a* and b* values between sample groups. A ΔE*_ab_ value greater than 3 was used as the threshold for a visually perceptible color difference under the applied measurement conditions [19].

### 2.4. Targeted Quantification of Free Amino Acids

Twenty-one free amino acids (FAAs) were quantified using targeted liquid chromatography–tandem mass spectrometry (LC–MS/MS). An aliquot of each endpoint sample was transferred into a 2 mL centrifuge tube, and 400 μL of 10% formic acid (≥98%; TCI) in methanol–water solution (1:1, *v*/*v*) was added for extraction. The mixture was vortexed for 30 s and centrifuged at 12,000 rpm for 5 min at 4 °C. The supernatant was diluted 10-fold with the extraction solution. Then, 100 μL of diluted supernatant was mixed with 100 μL of Trp-d3 internal standard solution (10 ng/mL, C/D/N Isotopes Inc., Canada), vortexed for 30 s, and filtered through a 0.22 μm membrane (Tianjin Jinteng Experimental Equipment Co., Ltd. (Tianjin, China) before analysis.

Chromatographic separation was performed using a Jasper HPLC system (SCIEX, Framingham, MA, USA) equipped with a ZORBAX Eclipse XDB-C18 column (4.6 × 150 mm; Agilent, Santa Clara, CA, USA). The injection volume was 5 μL, and the column temperature was maintained at 40 °C. The mobile phase consisted of 10% methanol in water containing 0.1% formic acid (A) and 50% methanol in water containing 0.1% formic acid (B). The gradient program was as follows: 0–6.5 min, 10–30% B; 6.5–7 min, 30–100% B; 7–18 min, 100% B; 18–18.5 min, 100–10% B; and 18.5–21 min, 10% B. The flow rate was 0.3 mL/min from 0 to 8 min and 0.4 mL/min from 8.5 to 21 min.

Mass spectrometric detection was performed using an AB SCIEX 4500MD mass spectrometer (SCIEX, Framingham, MA, USA) equipped with an electrospray ionization source in positive ion mode. The ion source temperature was 500 °C, the ion spray voltage was 5500 V, the collision gas was 6 psi, the curtain gas was 30 psi, and both nebulizer gas and auxiliary gas were set at 50 psi. Multiple reaction monitoring mode was used for data acquisition. FAA concentrations were calculated from the corresponding calibration curves and expressed as μg/mL.

For taste-associated chemical interpretation, the quantified FAAs were grouped into Sweet/Umami, Sour/Bitter, and Tasteless/Dull categories according to reported amino acid taste attributes and tea taste-related amino acid studies [12]. This grouping was used only to summarize taste-associated chemical patterns and was not considered a substitute for sensory evaluation.

### 2.5. Targeted Quantification of Flavonoids

Flavonoids were quantified by ultra-performance liquid chromatography–tandem mass spectrometry (UPLC–MS/MS) using a targeted panel of 35 authentic standards, comprising flavones, isoflavones, flavonols, flavonol glycosides, flavanols and flavanones. Each endpoint sample was extracted with 600 μL methanol in a 2-mL centrifuge tube. After vortexing for 60 s and ultrasonication for 15 min at room temperature, the extract was centrifuged at 12,000 rpm for 5 min at 4 °C. The supernatant was diluted fivefold with 80% methanol and passed through a 0.22-μm membrane filter before analysis.

Chromatographic separation was performed on an ACQUITY UPLC BEH C18 column (2.1 × 100 mm, 1.7 μm; Waters, Milford, MA, USA) using an ACQUITY UPLC system (Waters Corporation, Milford, MA, USA). The injection volume was 5 μL, and the column temperature was maintained at 40 °C. The mobile phase consisted of 0.1% formic acid in water (A) and methanol (B), with a flow rate of 0.25 mL/min. The gradient program was as follows: 0–1 min, 10% B; 1–3 min, 10–33% B; 3–10 min, 33% B; 10–15 min, 33–50% B; 15–20 min, 50–90% B; 20–21 min, 90% B; 21–22 min, 90–10% B; and 22–25 min, 10% B.

Mass spectrometric detection was performed on an AB5000 mass spectrometer (AB SCIEX, Marlborough, MA, USA) equipped with an electrospray ionization source operated in negative ion mode. The ion source temperature was 500 °C, the ion spray voltage was −4500 V, the collision gas was 6 psi, the curtain gas was 30 psi, and both nebulizer gas and auxiliary gas were set at 50 psi. Multiple reaction monitoring mode was used for data acquisition. Flavonoid concentrations were calculated using external calibration curves of the corresponding authentic standards, with correction for dilution and extraction volume, and expressed as μg/mL.

Of the 35 targeted flavonoids, 13 were not detected in any of the 18 samples. Baicalin and kaempferide were detected in only 2 and 3 samples, respectively, and were excluded because their limited detection coverage precluded reliable group-level comparisons. The remaining 20 flavonoids, each detected in at least 50% of the samples, were retained for downstream analysis and visualization. These compounds were classified as flavones and isoflavones, flavonols and flavonol glycosides, flavanols, or flavanones. Total quantified flavonoid content was calculated as the sum of the 20 retained compounds, and subclass contents were calculated by summing the compounds assigned to each class.

### 2.6. LC–MS/MS-Based Metabolomic Profiling

LC–MS/MS-based metabolomic profiling was performed to characterize a broader set of non-volatile metabolites beyond the targeted FAA and flavonoid assays. Samples from the three endpoint groups were thawed at 4 °C and vortexed for 1 min. An aliquot of each sample was transferred into a 2 mL centrifuge tube and concentrated to dryness. The dried residue was extracted with 500 μL of methanol (purity ≥99.0%, Fisher Scientific, Loughborough, UK), vortexed for 1 min, and centrifuged at 12,000 rpm for 10 min at 4 °C. The supernatant was transferred to a new centrifuge tube and dried again. The dried extract was reconstituted with 150 μL of 80% methanol–water containing 4 ppm 2-chloro-L-phenylalanine (purity, 98%; CAS no. 103616-89-3) as an internal standard, filtered through a 0.22 μm membrane, and transferred into an LC–MS vial. A pooled quality-control (QC) sample was prepared by mixing equal aliquots of the processed study samples and was used to monitor analytical stability during acquisition.

Chromatographic separation was performed on a Thermo Vanquish ultrahigh-performance liquid chromatography system equipped with an ACQUITY UPLC HSS T3 column (2.1 × 100 mm, 1.8 μm; Waters, Milford, MA, USA). The column temperature was maintained at 40 °C, the flow rate was 0.3 mL/min, and the injection volume was 2 μL. Data were acquired in both positive and negative electrospray ionization modes. In positive ion mode, the mobile phases were 0.1% formic acid in water (A) and 0.1% formic acid in acetonitrile (B). In negative ion mode, the mobile phases were 5 mM ammonium formate in water (A) and acetonitrile (B). The gradient program was as follows: 0–1 min, 8% B; 1–8 min, 8–98% B; 8–10 min, 98% B; 10–10.1 min, 98–8% B; and 10.1–12 min, 8% B.

Mass spectrometric detection was performed on a Q Exactive Focus mass spectrometer (Thermo Fisher Scientific, Bremen, Germany) equipped with an electrospray ionization source. The spray voltages were set at 3.50 kV in positive ion mode and −2.50 kV in negative ion mode. The sheath gas and auxiliary gas were set at 40 and 10 arbitrary units, respectively, and the capillary temperature was 325 °C. Full mass spectrometry (MS) scans were acquired over a mass-to-charge ratio (*m*/*z*) range of 100–1000 at a resolution of 70,000. MS/MS spectra were acquired in data-dependent acquisition mode using higher-energy collisional dissociation at a collision energy of 30 eV and a resolution of 17,500. The top three precursor ions were selected for fragmentation in each acquisition cycle, and dynamic exclusion was applied.

### 2.7. Metabolomic Data Preprocessing and Metabolite Annotation

Raw LC–MS/MS data were converted to mzXML format using MSConvert in ProteoWizard (version 3.0.8789). Peak detection, retention-time correction, peak alignment, and peak filling were performed using the XCMS package (version 3.12.0) in R (version 4.0.3) [20,21,22]. The main parameters were set as follows: bw = 2, ppm = 15, peakwidth = c(5, 30), mzwid = 0.015, mzdiff = 0.01, and method = “centWave”. The resulting data matrix contained feature ID, *m*/*z*, retention time, and peak intensity information for each sample.

QC-based signal correction was applied to reduce systematic analytical variation. Analytical reproducibility was evaluated using pooled QC samples, and features with a relative standard deviation greater than 30% in QC samples were removed before downstream analysis [23]. The retained feature matrix was used for multivariate analysis, differential metabolite screening, and metabolite annotation.

Metabolite annotation was based on accurate mass, adduct information, isotope distribution, and MS/MS fragmentation patterns. Annotation sources in the final dataset included BioDeepDB, mzCloud, MassBank of North America (MoNA), Global Natural Products Social Molecular Networking (GNPS), and MetaDNA; the Human Metabolome Database (HMDB) and the Kyoto Encyclopedia of Genes and Genomes (KEGG) identifiers were used for compound information and pathway mapping where available. All profiling annotations were assigned Level 2 and remained putative because they were not confirmed by matching both retention time and MS/MS spectra to authentic standards. Only MS/MS-annotated metabolites that passed quality filtering were retained for candidate metabolite screening and pathway analysis.

### 2.8. Multivariate Analysis, Candidate Metabolite Screening, and KEGG Pathway Analysis

Principal component analysis (PCA) was used to examine the overall distribution of metabolite profiles among SHt-D0, SFt-D7, and LFt-D7. Orthogonal partial least-squares discriminant analysis (OPLS-DA) provided VIP values for pairwise screening. Before multivariate analysis, metabolite intensities were autoscaled. PCA and pairwise OPLS-DA were performed using the ropls package (version 1.22.0) in R (version 4.0.3) [24]. One model with one predictive and one orthogonal component was constructed for each pairwise comparison. R^2^X (cum), R^2^Y (cum), and cross-validated Q^2^ (cum) were calculated, and 100 class-label permutations were performed.

For each pairwise comparison, Student’s *t*-tests were applied to the MS/MS-annotated metabolites. Metabolites with VIP > 1 and nominal *p* < 0.05 were retained as candidate differential metabolites. Benjamini–Hochberg false discovery rate (FDR) values were calculated as adjusted statistical information but were not used as a selection threshold. Higher and lower abundance refer to relative peak intensity in the first group compared with the second.

KEGG analysis summarized the pathway distribution of candidate differential metabolites. Metabolites were mapped to KEGG pathways and analyzed in MetaboAnalyst [25] using a hypergeometric enrichment test and degree-centrality topology analysis. The number of mapped metabolites, *p* value, FDR, and pathway impact were reported. Representative metabolites from tea-relevant pathways were grouped as amino acid/precursor, phenolic acid/phenylpropanoid, flavonoid-related, sugar/transport-related, or alkaloid-related.

### 2.9. Analysis of Alkaloid- and Phenolic Acid-Related Metabolites

Alkaloid- and phenolic acid-related metabolites were extracted from the MS/MS-annotated LC–MS/MS metabolomic profiling dataset according to compound annotations and chemical class information. Because these metabolites were obtained from profiling-derived peak intensity data rather than targeted absolute quantification, their abundances were expressed as relative peak abundances.

For heatmap visualization, the relative peak abundance of each metabolite was normalized across samples using row-wise Z-score transformation. To summarize class-level patterns, Z-score-normalized abundance values of metabolites within each chemical class were averaged at the sample level to generate class-level normalized abundance scores. These scores were used to compare the overall distribution patterns of alkaloid- and phenolic acid-related metabolites among SHt-D0, SFt-D7, and LFt-D7.

Representative alkaloid- and phenolic acid-related metabolites were selected to display compound-level patterns. Because the data were profiling-derived peak intensities, the comparisons describe relative abundance rather than absolute concentration or total class content.

### 2.10. Integrated Metabolite–Trait Association Analysis

Integrated metabolite–trait association analysis was performed using sample-level data to examine covariation between non-volatile metabolite modules and color- or taste-associated chemical traits. Four metabolite modules were defined according to compound class: FAA, quantified flavonoids (FLA), alkaloid-related metabolites (ALK), and phenolic acid-related metabolites (PHA). The chemical trait matrix included L*, a*, b*, C*_ab_, theaflavins, thearubigins, theabrownins, total FAAs, and total quantified flavonoid content.

Before analysis, metabolite and trait matrices were matched by sample name, and only common samples were retained. Variables with zero variance were removed. Missing values, if present, were imputed using the median value of the corresponding variable. Metabolite and trait variables were standardized before distance calculation and association analysis.

Mantel tests were performed using the linkET package (version 0.0.3) in R (version 4.0.3). Euclidean distance was used to calculate distance matrices for both metabolite modules and chemical traits, and Spearman’s method was used for Mantel correlation analysis. Mantel’s *r* and nominal *p* values represented the strength and statistical evidence of module-trait associations, respectively. These associations were interpreted as covariation patterns rather than evidence of causal relationships. Spearman correlation coefficients among chemical traits were calculated and displayed as the lower triangular correlation heatmap. For visualization, Mantel’s *r* values were grouped into weak (*r* < 0.2), moderate (0.2 ≤ *r* < 0.4), and strong (*r* ≥ 0.4) associations, and Mantel’s *p* values were grouped to indicate statistical significance.

### 2.11. Statistical Analysis

Unless otherwise indicated, analyses used six samples per group. Data are presented as mean ± standard deviation (s.d.). Group differences among SHt-D0, SFt-D7, and LFt-D7 were assessed by one-way analysis of variance (ANOVA) followed by Tukey’s honestly significant difference (HSD) test. *p* < 0.05 was considered statistically significant. Different lowercase letters in the figures denote significant group differences.

## 3. Results

### 3.1. Physicochemical Properties and Chromatic Characteristics

**Figure 1:**
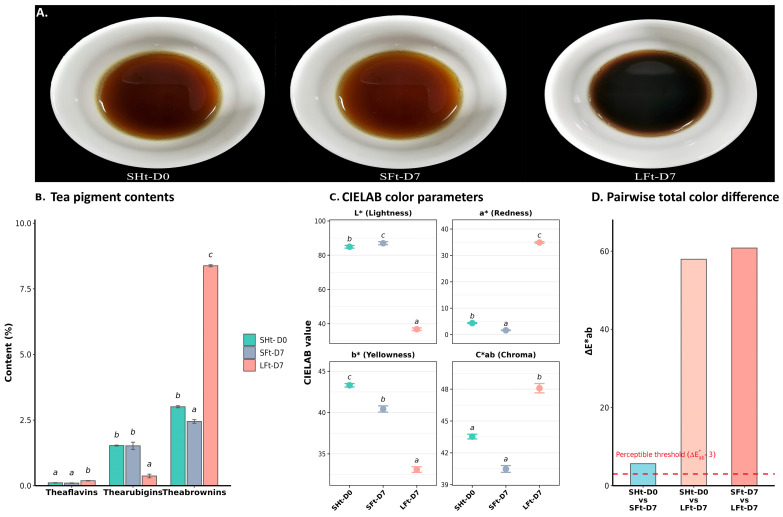
Endpoint color and pigment characteristics of Fu tea samples prepared through different routes. (**A**) Representative infusion images of SHt-D0, SFt-D7 and LFt-D7. (**B**) Contents of theaflavins, thearubigins and theabrownins. (**C**) CIELAB color parameters, including L*, a*, b* and C*_ab_. (**D**) Pairwise total color difference ΔE*_ab_; the dashed line indicates the perceptible threshold of ΔE*_ab_ = 3. Data are shown as mean ± s.d. (*n* = 6). Different lowercase letters indicate significant differences among groups for the same index (one-way ANOVA followed by Tukey’s HSD test, *p* < 0.05).

The three endpoint groups differed in infusion color (Figure 1A). SHt-D0 and SFt-D7 produced relatively bright reddish-yellow infusions, whereas LFt-D7 was dark reddish-brown.

The measured tea pigment profile was consistent with this visual contrast (Figure 1B). Compared with SHt-D0 and SFt-D7, LFt-D7 showed significantly higher theaflavin and theabrownin contents, together with a marked decrease in thearubigins. Theabrownin content increased from 3.01% in SHt-D0 to 8.38% in LFt-D7, whereas SFt-D7 showed a lower theabrownin content than SHt-D0. The darker appearance of LFt-D7 coincided with higher theabrownin and lower thearubigin contents, while the remaining pigment fractions did not change in the same direction.

CIELAB analysis quantified the magnitude and direction of these chromatic shifts (Figure 1C). LFt-D7 was characterized by lower L* and higher a*, indicating reduced lightness and stronger redness. Its b* value decreased, whereas C*_ab_ increased, reflecting changes in both the yellow-blue axis and overall chromatic intensity. By contrast, SFt-D7 remained closer to SHt-D0 across L*, a*, b*, and C*_ab_, suggesting that chromatic remodeling was much more pronounced in the liquid-state fermented endpoint sample than in the aqueous extract of solid-state fermented tea.

The pairwise total color difference further confirmed this separation (Figure 1D). The ΔE*_ab_ value between SHt-D0 and SFt-D7 was 5.64, indicating a perceptible but moderate color difference. In contrast, the ΔE*_ab_ values for SHt-D0 versus LFt-D7 and SFt-D7 versus LFt-D7 reached 57.95 and 60.82, respectively, far exceeding the perceptibility threshold of 3. Thus, LFt-D7 showed the largest visual and instrumental color difference among the three endpoint groups.

### 3.2. LC–MS/MS Profiling of Preparation-Route-Associated Metabolic Variation

**Figure 2:**
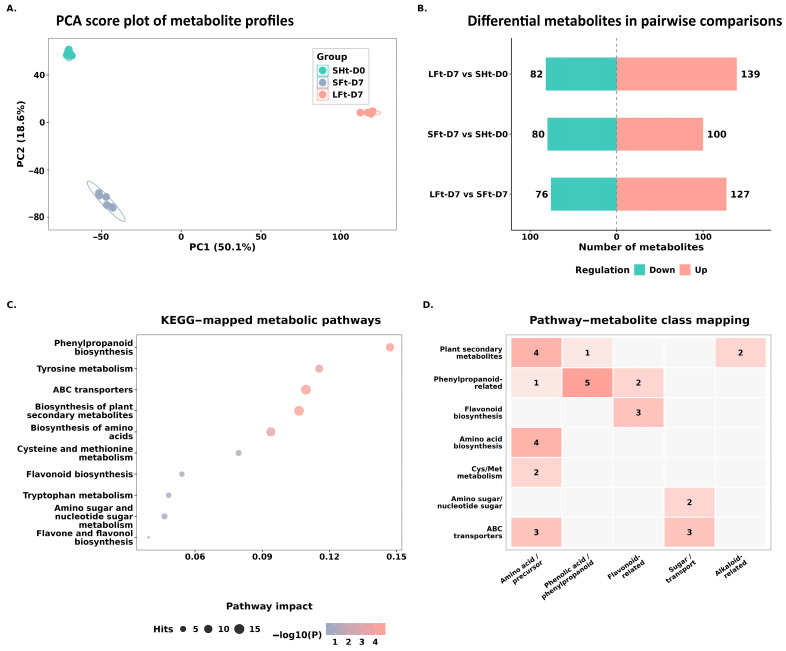
LC–MS/MS-based metabolomic profiling of the three endpoint groups. (**A**) PCA of metabolomic profiles from SHt-D0, SFt-D7 and LFt-D7. (**B**) Numbers of annotated metabolites meeting the VIP > 1 and nominal *p* < 0.05 screening criteria in each pairwise comparison; higher or lower abundance is defined relative to the first group. (**C**) KEGG pathway enrichment and topology overview of screened metabolites. (**D**) Pathway–metabolite class mapping of representative metabolites from selected tea-relevant pathways. Numbers within the cells indicate the numbers of representative metabolites assigned to each pathway–metabolite class combination.

LC–MS/MS profiling was used to examine non-volatile composition across the three endpoint groups. After quality filtering, 447 unique Level 2 putative annotations were retained, including 226 in positive-ion mode and 221 in negative-ion mode. PCA separated SHt-D0, SFt-D7, and LFt-D7, with PC1 and PC2 accounting for 50.1% and 18.6% of the total variance, respectively (Figure 2A). Replicates clustered within groups, and LFt-D7 occupied a distinct region of the score plot relative to SHt-D0 and SFt-D7.

Pairwise OPLS-DA models were used to calculate VIP values. The R^2^X (cum), R^2^Y (cum), and Q^2^ (cum) values were 0.680, 1.000, and 0.994 for LFt-D7 versus SHt-D0; 0.398, 0.999, and 0.927 for SFt-D7 versus SHt-D0; and 0.644, 1.000, and 0.992 for LFt-D7 versus SFt-D7, respectively. In the 100-permutation tests, all permuted-model Q^2^ values were lower than the corresponding original-model Q^2^ values.

Using VIP > 1 and nominal *p* < 0.05 as screening criteria, 221 annotated metabolites met the criteria for LFt-D7 versus SHt-D0; 139 had higher and 82 had lower relative abundance in LFt-D7 (Figure 2B). For SFt-D7 versus SHt-D0, 180 metabolites met the criteria, including 100 with higher and 80 with lower relative abundance in SFt-D7. The LFt-D7 versus SFt-D7 comparison yielded 203 metabolites, of which 127 were higher and 76 were lower in LFt-D7.

KEGG mapping summarized the pathway annotations of the screened metabolites (Figure 2C). The selected tea-relevant pathways included plant secondary-metabolite biosynthesis, phenylpropanoid biosynthesis, flavonoid biosynthesis, flavone and flavonol biosynthesis, amino acid biosynthesis and metabolism, carbohydrate metabolism, and ABC transporters. Phenylpropanoid biosynthesis had relatively high pathway impact, whereas plant secondary-metabolite biosynthesis and ABC transporters contained more mapped metabolites.

Pathway–metabolite mapping placed phenolic acid- and flavonoid-related metabolites in phenylpropanoid and flavonoid pathways, amino acid-related metabolites in amino acid biosynthesis and cysteine/methionine metabolism, and sugar- or transport-related metabolites in amino sugar/nucleotide sugar metabolism and ABC transporter annotations (Figure 2D).

### 3.3. Preparation-Route-Associated Differences in Free Amino Acids

**Figure 3:**
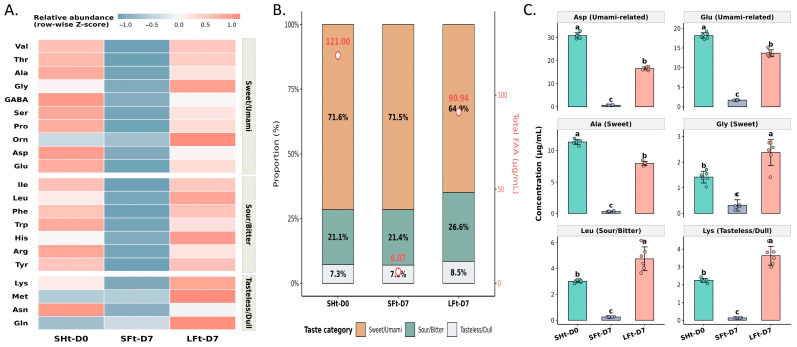
Targeted free amino acid profiling reveals divergent amino acid-related chemical traits. (**A**) Heatmap of 21 quantified free amino acids (FAAs) grouped by taste-associated categories. Colors indicate row-wise Z-score-normalized abundance. (**B**) Relative proportions of FAA taste categories and total FAA content across the three endpoint groups. (**C**) Contents of six representative FAAs. Bars show mean values, error bars indicate s.d., and points indicate individual samples (*n* = 6). Different lowercase letters indicate significant differences among groups for each amino acid (one-way ANOVA followed by Tukey’s HSD test, *p* < 0.05).

FAAs are important non-volatile taste-associated compounds in tea infusions. To characterize FAA variation across the three endpoint groups, 21 FAAs were quantified and grouped according to reported taste-associated attributes into Sweet/Umami, Sour/Bitter, and Tasteless/Dull categories (Figure 3A). The heatmap showed strong depletion of most FAAs in SFt-D7, whereas LFt-D7 retained substantially higher levels of many amino acids. This contrast separated the two routes at the level of soluble FAA composition.

The total FAA pool quantified this difference (Figure 3B). SHt-D0 contained 121.00 μg/mL total FAAs, whereas SFt-D7 decreased sharply to 6.07 μg/mL. In contrast, LFt-D7 retained 90.94 μg/mL, indicating a substantially larger detectable FAA pool than SFt-D7. After assignment of Asp and Glu to the Sweet/Umami category, Sweet/Umami-related FAAs represented the largest taste-associated category in all three endpoint groups (71.6% in SHt-D0, 71.5% in SFt-D7, and 64.9% in LFt-D7). Sour/Bitter FAAs accounted for 21.1%, 21.4%, and 26.6%, respectively, whereas Tasteless/Dull FAAs accounted for 7.3%, 7.0%, and 8.5%. Therefore, the strongest route-associated difference was reflected in the size of the FAA pool rather than a complete restructuring of the taste-category proportions.

Compound-level comparisons of six representative FAAs further clarified the pattern (Figure 3C). Asp, Glu, and Ala were strongly depleted in SFt-D7 but remained at higher levels in LFt-D7. By contrast, Gly, Leu, and Lys reached their highest detected contents in LFt-D7. These compound-specific responses indicate that FAA variation was not a simple uniform decrease across all amino acids; instead, individual FAAs showed distinct route-associated retention or enrichment patterns.

Overall, the FAA profiles separated the two routes at the level of soluble amino acid composition. SFt-D7 was characterized by extensive depletion of the soluble FAA pool, whereas LFt-D7 retained a much larger portion of the baseline amino acid pool and showed higher levels of selected individual amino acids. As sensory testing was not performed, these results should be interpreted as differences in taste-associated chemical substrates rather than direct evidence of perceived taste differences.

### 3.4. Subclass-Specific Differences in Targeted Flavonoids

**Figure 4:**
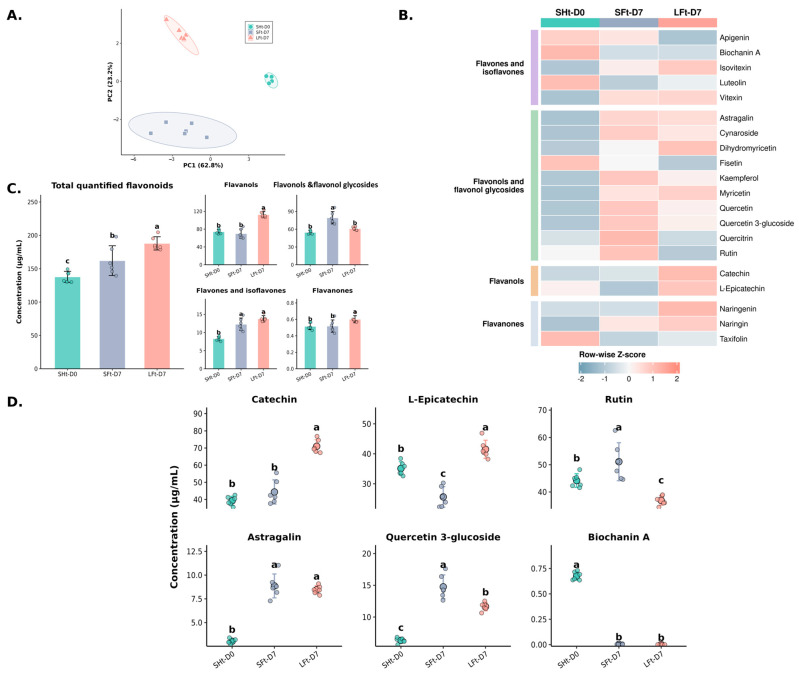
Targeted flavonoid profiling reveals subclass-specific remodeling of quantified flavonoids. (**A**) PCA of 20 quantified flavonoids. (**B**) Heatmap of quantified flavonoids grouped by chemical subclass; colors indicate row-wise Z-score-normalized abundance. (**C**) Total quantified flavonoid content and subclass-level contents. (**D**) Compound-level profiles of six representative flavonoids. In (**C**,**D**), points represent individual samples, error bars indicate s.d., and different lowercase letters indicate significant differences among groups (one-way ANOVA followed by Tukey’s HSD test, *p* < 0.05).

Targeted flavonoid profiling was next performed to assess phenolic changes among the three endpoint groups. Of the 35 targeted flavonoids, 20 were detected in at least 50% of the samples and retained for subsequent analysis. PCA based on these compounds separated SHt-D0, SFt-D7 and LFt-D7, with PC1 and PC2 accounting for 62.8% and 23.2% of the total variance, respectively (Figure 4A). SHt-D0 was separated from both fermented endpoint groups primarily along PC1, whereas SFt-D7 and LFt-D7 were distinguished along PC2.

The subclass-annotated heatmap revealed compound-specific remodeling within the flavonoid pool (Figure 4B). LFt-D7 was enriched in the major flavanols catechin and L-epicatechin. Several flavonols and flavonol glycosides, including astragalin, quercetin 3-glucoside, quercetin, myricetin, kaempferol, and dihydromyricetin, were higher in SFt-D7 and/or LFt-D7, depending on the compound, whereas other flavonoids declined after fermentation.

Total quantified flavonoid content differed significantly among the three groups (Figure 4C). LFt-D7 showed the highest total quantified flavonoid content, followed by SFt-D7, whereas SHt-D0 showed the lowest level. At the subclass level, flavanols contributed strongly to the elevated quantified flavonoid content in LFt-D7, while flavonols and flavonol glycosides were more closely associated with SFt-D7. The aqueous extract of solid-state fermented tea therefore displayed a stronger flavonol and flavonol–glycoside signature, while the liquid-state fermented infusion showed a flavanol-rich profile with the highest total quantified flavonoid content.

Representative compound-level profiles further supported these subclass-level differences (Figure 4D). Catechin and L-epicatechin were enriched in LFt-D7, whereas rutin reached its highest level in SFt-D7 and its lowest level in LFt-D7. Astragalin and quercetin 3-glucoside increased after solid-state fermentation and remained above baseline in LFt-D7. In contrast, Biochanin A was abundant in SHt-D0 but was nearly depleted in both fermented endpoint groups. These representative compounds reinforce the route-associated flavonoid pattern, with LFt-D7 characterized by flavanol enrichment and the highest total quantified flavonoid content, and SFt-D7 by a stronger flavonol and flavonol–glycoside signature.

### 3.5. Relative Profiling of Alkaloid- and Phenolic Acid-Related Metabolites

To extend the analysis beyond FAAs and flavonoids, alkaloid- and phenolic acid-related metabolites were extracted from the MS/MS-annotated LC–MS/MS profiling dataset. Because these compounds were obtained from profiling-derived peak intensity data rather than targeted absolute quantification, they are described here as relative peak abundances or class-level normalized scores.

**Figure 5:**
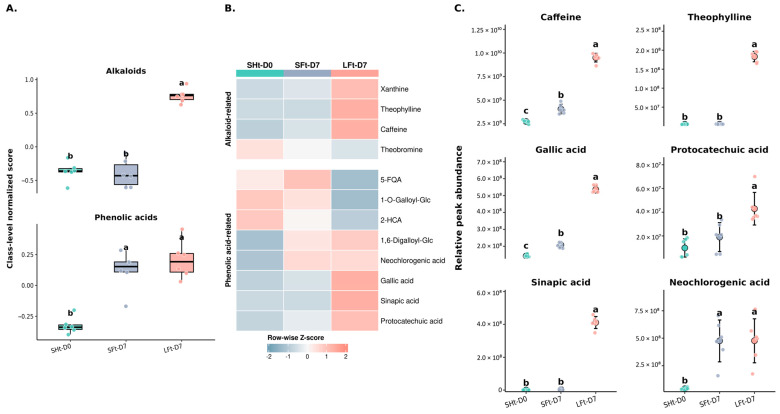
Relative profiling of alkaloid- and phenolic acid-related metabolites. (**A**) Class-level normalized scores of alkaloid-related and phenolic acid-related metabolites across SHt-D0, SFt-D7 and LFt-D7. Boxplots summarize sample-level class scores, and points represent individual samples. (**B**) Heatmap of representative alkaloid- and phenolic acid-related metabolites based on row-wise Z-score-normalized relative peak abundance. (**C**) Relative peak abundances of six representative metabolites, including caffeine, theophylline, gallic acid, protocatechuic acid, sinapic acid and neochlorogenic acid. Points represent individual samples, and error bars indicate s.d. Different lowercase letters indicate significant differences among groups for the same metabolite or metabolite class (one-way ANOVA followed by Tukey’s HSD test, *p* < 0.05).

At the class level, alkaloid-related metabolites showed a clear route-associated pattern (Figure 5A). LFt-D7 had markedly higher class-level normalized scores than SHt-D0 and SFt-D7, indicating relative enrichment of alkaloid-related metabolites in the liquid-state fermented infusion. Phenolic acid-related metabolites also differed among the three endpoint groups, with fermented samples generally showing higher class-level normalized scores than SHt-D0. However, the separation between SFt-D7 and LFt-D7 was less pronounced for phenolic acid-related metabolites than for alkaloid-related metabolites.

The class-annotated heatmap further resolved metabolite-specific differences within these two classes (Figure 5B). Representative alkaloid-related metabolites, including xanthine, theophylline, caffeine, and theobromine, showed higher relative abundance in LFt-D7 than in SHt-D0 and SFt-D7. Phenolic acid-related metabolites displayed a more heterogeneous pattern. Gallic acid, protocatechuic acid, sinapic acid, and neochlorogenic acid were relatively enriched in LFt-D7, whereas 5-O-feruloylquinic acid and 2-hydroxycinnamic acid showed lower relative abundance. The phenolic acid-related class was therefore heterogeneous, with enrichment restricted to selected compounds.

The relative peak abundances of six representative metabolites supported the class-level and heatmap observations (Figure 5C). Among alkaloid-related metabolites, caffeine and theophylline showed higher relative abundance in LFt-D7 than in SHt-D0 and SFt-D7. Among phenolic acid-related metabolites, gallic acid, protocatechuic acid, and sinapic acid were also relatively enriched in LFt-D7, whereas neochlorogenic acid increased in both fermented endpoint groups compared with SHt-D0. These relative-abundance profiles identified alkaloid- and phenolic acid-related metabolites as additional features distinguishing LFt-D7 in the peak-intensity-based dataset.

### 3.6. Integrated Associations Between Metabolite Modules and Color- and Taste-Associated Chemical Traits

**Figure 6:**
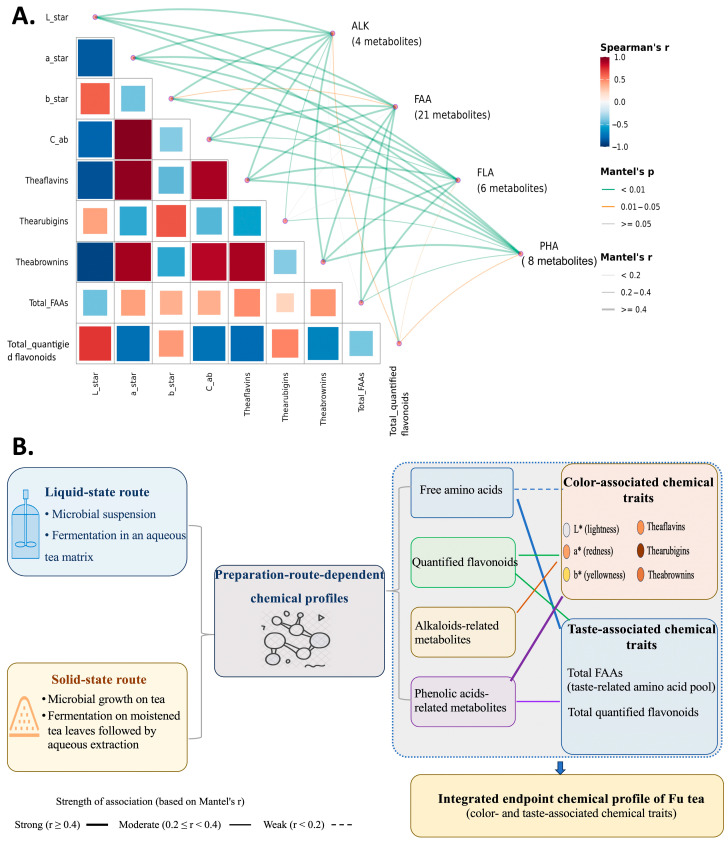
Integrated associations between metabolite modules and endpoint chemical traits. (**A**) Mantel test-based link heatmap integrating four non-volatile metabolite modules with color- and taste-associated chemical traits. The metabolite modules include free amino acids (FAA), quantified flavonoids (FLA), alkaloid-related metabolites (ALK) and phenolic acid-related metabolites (PHA). The lower triangular matrix shows Spearman correlations among endpoint traits, including CIELAB color parameters, tea pigment indices, total FAAs and total quantified flavonoid content. Link color denotes Mantel’s *p* value, and link width denotes Mantel’s *r*. (**B**) Association-based schematic summarizing the coordinated endpoint chemical differences associated with the two preparation routes.

To integrate the metabolite-level differences described above with color- and taste-associated chemical traits, a Mantel test-based link heatmap was constructed using four non-volatile metabolite modules: free amino acids (FAA), quantified flavonoids (FLA), alkaloid-related metabolites (ALK), and phenolic acid-related metabolites (PHA) (Figure 6A). These modules were analyzed together with physicochemical and chromatic traits, including CIELAB color parameters, tea pigment indices, total FAAs, and total quantified flavonoid content. The lower triangular Spearman correlation matrix showed structured relationships among these traits. L* was negatively associated with several redness- and pigment-related variables, whereas a*, C*_ab_, theaflavins, and theabrownins clustered with the chromatic separation observed among the three endpoint groups.

The Mantel links indicated that the four metabolite modules were associated with different sets of color- and taste-associated chemical traits. The FAA module showed the strongest association with total FAAs (Mantel’s *r* = 0.893, *p* = 0.001), consistent with the targeted FAA profiles and serving as an internal consistency check for the amino acid module. PHA showed multiple moderate-to-strong associations with chromatic parameters and tea pigment indices, suggesting that phenolic acid-related metabolites covaried closely with color-associated chemical variation. ALK was also associated with several pigment- and color-associated traits, including theabrownins, theaflavins, L*, and a*. The FLA module was connected with both chromatic traits and total quantified flavonoid content, reflecting the close relationship between quantified flavonoid variation and the broader non-volatile phenolic profile of the three endpoint groups. These module–trait associations indicate that the chemical differences among the three endpoint groups were distributed across multiple metabolite classes rather than being confined to a single compound group.

Based on the Mantel associations, targeted quantification and relative-abundance profiles, Figure 6B was used to summarize the coordinated endpoint chemical differences associated with the two preparation routes. SFt-D7 was characterized by extensive depletion of the FAA pool and a stronger flavonol and flavonol–glycoside signature. In contrast, LFt-D7 showed pronounced chromatic alteration, partial retention of the baseline FAA pool, higher total quantified flavonoid content with flavanol enrichment, and relative enrichment of selected alkaloid- and phenolic acid-related metabolites. This integrated pattern summarizes preparation-route-associated differences across non-volatile metabolite modules and the linked color- and taste-associated chemical traits, without implying direct causal relationships.

## 4. Discussion

### 4.1. Color and Pigment Differences Between Preparation Routes

Color provided the clearest visual distinction between the two fermented endpoint products. LFt-D7 developed a dark reddish-brown infusion, whereas SFt-D7 remained closer to the bright reddish-yellow appearance of SHt-D0. The lower L*, higher a*, and large ΔE*_ab_ values of LFt-D7 confirmed the instrumental color shift. Comparable differences in CIELAB parameters and pigment composition have been reported across tea manufacturing processes, cultivars, and *E. cristatum*-fermented teas [26,27].

The pigment profile provides a chemical context for this phenotype. Tea infusion color is generated by a heterogeneous set of catechin-derived oxidation, condensation and polymerization products rather than by a single pigment fraction. Theaflavins are relatively well-defined oxidation products, whereas thearubigins and theabrownins are chemically complex mixtures whose composition varies with oxidation and fermentation conditions [9,10]. Recent reviews further describe theabrownins as water-soluble brown pigment mixtures formed mainly through oxidative polymerization of tea polyphenols, while emphasizing that their molecular heterogeneity remains difficult to resolve [28,29]. In LFt-D7, higher measured theabrownins occurred together with lower thearubigins and pronounced darkening. This pattern is consistent with redistribution toward darker pigment-related fractions, although the spectrophotometric indices used here define operational fractions and cannot identify the colored molecules themselves.

Studies of aqueous *E. cristatum* fermentation provide a useful comparison. During liquid-state fermentation of instant dark tea, galloylated catechins and free amino acids declined, whereas nongalloylated catechins, alkaloids, thearubigins and theabrownins increased [7]. Submerged fermentation also altered the volatile and non-volatile composition of dark tea infusions, with the direction of change depending on the starting tea matrix [8,30,31]. In the present endpoint dataset, catechin-derived intermediates and enzyme activities were not measured.

SFt-D7 showed a smaller color difference from SHt-D0. Solid-state fermentation studies have reported changes in catechins, flavonoids, phenolic acids and tea pigments, with patterns varying across fermentation stages, moisture conditions, substrate structures and extraction procedures [5,6,32]. Related work has also documented microbial bioconversion of major chemical constituents during dark-tea processing [33]. In the present study, SFt-D7 was extracted after fermentation, whereas LFt-D7 was fermented directly in the aqueous phase. The observed contrast therefore pertains to the complete preparation routes, which also differed in thermal history and compound recovery.

### 4.2. Divergent Free Amino Acid Profiles Between Preparation Routes

The two fermented endpoints differed markedly in soluble FAAs. SFt-D7 contained a much smaller FAA pool, whereas LFt-D7 retained more FAAs, although still less than SHt-D0. Comparable changes have been reported in microbially processed dark tea, other tea-processing systems [14,34], and aqueous *E. cristatum* fermentation [7]. A paired study of Fu brick tea before and after flowering reported a mean 38.24% decrease in total FAAs, with all 19 quantified amino acids declining [35].

This contrast probably reflects more than one process. In the solid matrix, amino acids may have been consumed or transformed through microbial metabolism, retained within the leaf structure, or recovered less efficiently during extraction. Solid-state fermentation of dark tea has previously been associated with substantial changes in amino acid-related metabolites and taste characteristics [6]. In the liquid route, amino acids were already dissolved in the aqueous matrix and may have remained more accessible in the final infusion. Protein or peptide hydrolysis, compound-specific stability, and differences in heating and extraction history may also have affected the measured profiles. The available endpoint data do not separate these contributions.

The six representative FAAs did not change uniformly. Asp, Glu and Ala remained comparatively abundant in LFt-D7 but were strongly reduced in SFt-D7, while Gly, Leu and Lys reached their highest measured contents in LFt-D7. Each amino acid is likely to reflect a different balance among release, uptake, conversion, degradation and extraction. Amino acids and related metabolites have previously been associated with mellow, umami, bitter and other taste attributes in Fu brick tea [4]. Related studies have also connected changes in aspartic acid, glutamic acid and catechin-related compounds with instrumental taste responses [36], and variations in “golden flora” abundance with liquor color, metabolite profiles and astringency-related sensory quality [37].

### 4.3. Subclass-Specific Remodeling of Phenolic and Alkaloid-Related Metabolites

Phenolic changes differed among subclasses. LFt-D7 had the highest total quantified flavonoid content and was distinguished by flavanol enrichment, especially catechin and L-epicatechin. SFt-D7 showed a stronger flavonol and flavonol–glycoside signature, typified by rutin and by elevated astragalin and quercetin 3-glucoside. The two preparations differed in both total flavonoid content and phenolic subclass composition.

The flavanol-rich profile of LFt-D7 is consistent with previous observations from aqueous *E. cristatum* fermentation. An et al. [7] reported decreases in galloylated catechins and increases in nongalloylated catechins during liquid-state fermentation. The transformation of epigallocatechin gallate by *E. cristatum* has also been shown to produce gallic acid and epigallocatechin as major intermediates [38], while fermentation of black tea by this fungus altered catechin- and caffeine-related metabolic features [39]. These findings make fungal conversion of catechin-related compounds a plausible explanation for part of the LFt-D7 pattern. Differences in extraction, retention and non-enzymatic reactions may also have contributed.

Phenolic acids and flavonoids also respond to solid-state *E. cristatum* fermentation, with degradation, glycosylation, deglycosylation, methylation, and oxidative polymerization among the proposed reactions [5]. Inoculated solid-state fermentation of Pu-erh tea has likewise been associated with changes in major chemical components and flavor-related attributes [32], and flavonol glycosides can undergo polyphenol oxidase-dependent conversion during tea processing [40]. The higher relative levels of several flavonol glycosides in SFt-D7 may reflect release from the leaf matrix, chemical conversion, stability, and extraction recovery.

LFt-D7 also showed higher relative peak-intensity signals for caffeine, theophylline, gallic acid, protocatechuic acid, and sinapic acid. Processing-related variation in purine alkaloids, phenolic acids, and catechin-derived metabolites has been reported during liquid fermentation and conventional tea manufacture [7,33,34,39], and fungal starter species influence flavonoid-, phenylpropanoid-, pigment-, and caffeine-related profiles in liquid-state fermented instant dark tea [41]. Metabolite changes associated with bitterness and astringency have also been reported during pile fermentation of dark tea [42].

### 4.4. Integrated Endpoint Variation and Interpretive Limits

The Mantel analysis related the FAA, FLA, ALK, and PHA modules to pigment indices, CIELAB parameters, total FAAs, and total quantified flavonoid content. Similar relationships among microbial communities, metabolites and color- or taste-associated properties have been reported in Fu brick teas produced under different fermentation conditions, from different raw materials and across production regions [43,44,45]. Because total FAAs and total quantified flavonoid content were calculated from variables included in the FAA and FLA modules, respectively, these two associations are not independent.

PHA and ALK covaried with several pigment and color variables, showing that chromatic variation occurred alongside broader changes in non-volatile composition. Some associations may also reflect the separation of the three endpoint groups. The two routes produced distinct soluble endpoint profiles: LFt-D7 combined stronger chromatic development with greater FAA retention and a flavanol-rich profile, whereas SFt-D7 contained fewer FAAs and had a stronger flavonol and flavonol–glycoside signature. Previous studies have documented changes in these compound classes during liquid- or solid-state *E. cristatum* fermentation [5,6,7,8,32,44]. The present study extends that work by comparing the final products of both routes with the same analytical workflow.

The contrasting profiles may inform the choice of preparation route for fermented infusions, soluble ingredients, or extracted solid-state products. Such choices will depend on the starting tea, intended product form, and desired color and composition; they cannot be reduced to a general ranking of product quality. Processing format and raw material grade have likewise been shown to influence the metabolomic and sensory properties of Fu tea [45,46,47].

This endpoint design captures the combined outcome of the complete preparation routes, including differences in fermentation matrix, thermal history, extraction efficiency and microbial transformation, and does not isolate fermentation mode as a single factor. The mechanistic explanations therefore remain provisional pending enzyme activity measurements or controlled biotransformation experiments; similarly, as sensory evaluation was not included, the taste-associated classifications and metabolite–trait associations describe compositional patterns whose sensory relevance remains to be established. Time-resolved metabolomics has shown that metabolite trajectories vary across fermentation stages and fungal systems [48]. Future work combining temporal sampling, matched thermal and extraction controls, measurements of microbial dynamics and enzyme activity, and quantitative sensory or instrumental taste analysis could resolve these effects and determine whether the compositional differences translate into perceived product quality [37,49].

## 5. Conclusions

Using the same batch of tea and the same 7-day fermentation period, liquid-state fermentation and solid-state flowering followed by aqueous extraction produced clearly different soluble products. LFt-D7 developed a darker reddish-brown color and retained a larger free-amino-acid pool, whereas SFt-D7 showed greater amino acid depletion and a stronger flavonol and flavonol–glycoside pattern. These contrasts reflect the complete preparation processes, including differences in fermentation matrix, heat treatment and extraction, and cannot be attributed to fermentation mode alone. Comparing the two product formats under the same analytical framework shows that route selection involves different outcomes in color and soluble composition. Sensory evaluation and matched, time-resolved experiments are needed to determine how these chemical differences arise and whether they influence perceived product quality.

## Data Availability

The data supporting the findings of this study are provided in the article. The ITS sequence generated in this study has been deposited in GenBank under accession number PZ742006. Subject to institutional approval, the fungal isolate used in this study may be made available by the corresponding author upon reasonable request. The original contributions presented in this study are included in the article and Supplementary Material. The ITS sequence generated in this study has been deposited in GenBank under accession number PZ742006. Subject to institutional approval, the fungal isolate used in this study may be made available by the corresponding author upon reasonable request. Further inquiries can be directed to the corresponding author.

## Supplementary Materials

The following supporting information can be downloaded at: https://www.mdpi.com/article/10.3390/foods15152719/s1, Figure S1: Representative surface morphology of *E. cristatum* isolate EC-JW01 on PDA after incubation at 28 °C for 5 days.

## Abbreviations

The following abbreviations are used in this manuscript:

SHt-D0	unfermented sterilized tea infusion
SFt-D7	aqueous extract of tea after 7 d of solid-state fermentation
LFt-D7	tea infusion after 7 d of liquid-state fermentation
FAA	free amino acid
FLA	quantified flavonoid module
ALK	alkaloid-related metabolite module
PHA	phenolic acid-related metabolite module
